# Identifying the visual and linguistic sources of reading efficiency: A comparison of individual differences among deaf and hearing readers

**DOI:** 10.1167/jov.26.6.2

**Published:** 2026-06-01

**Authors:** Elizabeth R. Schotter, Sara Milligan, Frances G. Cooley, Karen Emmorey

**Affiliations:** 1 Department of Psychology, University of South Florida, Tampa, FL, USA; 2 Department of Liberal Studies, National Technical Institute for the Deaf, Rochester Institute of Technology, Rochester, NY, USA; 3 School of Speech, Language, and Hearing Sciences, San Diego State University, San Diego, CA, USA

**Keywords:** reading efficiency, reading spans, individual differences, deaf readers

## Abstract

Reading is a task that engages the vision–language interface, and the efficiency of this process may be related to how much of the text readers use to process visual and/or linguistic information during a fixation (i.e., their *reading span size*). Compared with typically hearing readers, deaf signers both read more efficiently (i.e., faster with equivalent comprehension) and have wider reading spans, suggesting a critical role for span size in reading efficiency. Using a linear regression analysis, we assessed the effects of two different span sizes (the *word identification span*, within which readers extract linguistic information, and the *perceptual span*, within which they extract the visuospatial layout) and reading comprehension ability on reading rate for deaf signers (*n* = 50) and hearing non-signers (*n* = 109). For all readers, those with a larger word identification span read faster, but this relationship was weaker for the perceptual span, emphasizing the importance of parafoveal linguistic processing for reading speed. The effect of the word identification span was stronger for the deaf group, and only deaf signers showed a positive relationship between reading comprehension ability and reading rate (and word identification span size). These findings suggest that reading efficiency (exemplified by deaf signers) derives from an ability to extract linguistic information far from fixation in a way that supports both reading speed and comprehension.

## Introduction

To read successfully, a person must visually perceive words, allocate attention to recognize their meanings, and plan eye movements to the next location in the text (Rayner, Schotter, Masson, Potter, & Treiman, 2016). But, it is unclear which of the former two processes (visual or linguistic processing) has a greater effect on the latter (oculomotor control decisions that determine reading speed). Any change early in life to a component brain system that supports these functions may impact the way that the entire reading system performs—detrimentally but also beneficially—and investigating these adaptations can help us understand the vision–language interface as it applies to all readers. For example, it is often assumed that deaf people read poorly, but this is a common misconception, and the current reading abilities of deaf people are within the average range for age-based norms (Mayer, Trezek, & Hancock, 2021). Nevertheless, this misconception persists because of the assumption that reduced auditory input prohibits them from “sounding out” words (Aparicio, Gounot, Demont, & Metz-Lutz, 2007), a process that is beneficial for hearing readers (Castles, Rastle, & Nation, 2018; Leinenger, 2014; Milligan & Schotter, 2024). However, despite not automatically activating robust sound-based representations while reading (Bélanger, Mayberry, & Rayner, 2013; Costello, Caffarra, Fariña, Duñabeitia, & Carreiras, 2021; Fariña, Duñabeitia, & Carreiras, 2017; Friesen & Joanisse, 2012; Hirshorn, Dye, Hauser, Supalla, & Bavelier, 2015; Mayberry, del Giudice, & Lieberman, 2011; McQuarrie & Parrila, 2008; Peleg, Ben-Hur, & Segal, 2020; Sehyr & Emmorey, 2022), many deaf individuals do read successfully (e.g., Mayer et al., 2021; Sehyr & Emmorey, 2022). Furthermore, reading comprehension is higher for deaf signers compared with deaf non-signers and is positively related to sign language skills (Tomazin, Radošević, & Hrastinski, 2025; Zhang, Ke, Yang, & Anglin-Jaffe, 2024). In fact, some deaf people—particularly early sign language users—read *more* efficiently than hearing people (i.e., faster with equivalent comprehension) (Bélanger, Lee, & Schotter, 2018; Bélanger, Slattery, Mayberry, & Rayner, 2012; Cooley et al., 2025a, Cooley, Emmorey, Saunders, & Schotter, 2025b; Emmorey et al., 2025; Schotter et al., 2024; Stringer, Cooley, Saunders, Emmorey, & Schotter, 2024; see also Bélanger & Rayner, 2015; Emmorey, & Lee, 2021). One way they may overcome, or circumvent, the benefits that phonological representations afford to hearing readers is by distributing visual attention more broadly (Bavelier et al., 2000; Bavelier & Neville, 2002; Dye & Bavelier, 2013). To examine the vision–language interface in reading, and how that might impact reading efficiency, we examined the size of a person's *reading span* (i.e., the area from which information is extracted from the text) (Schotter et al., 2024), which is larger for faster compared with slower hearing readers (Rayner, Slattery, & Bélanger, 2010), and is larger for deaf early signers compared with hearing non-signers with equivalent reading abilities (Bélanger et al., 2012; Bélanger et al., 2018; Emmorey et al., 2025; Liu, Chen, Tong, & Su, 2021; Schotter et al., 2024; Stringer et al., 2024). More specifically, we investigated the contributions of two different types of spans—*perceptual span* (used to extract visuospatial information from the text) and *word identification span* (used to extract linguistic information from the text) (Schotter et al., 2024)—in determining reading rate for deaf signers and hearing non-signers.

**Figure 1.**
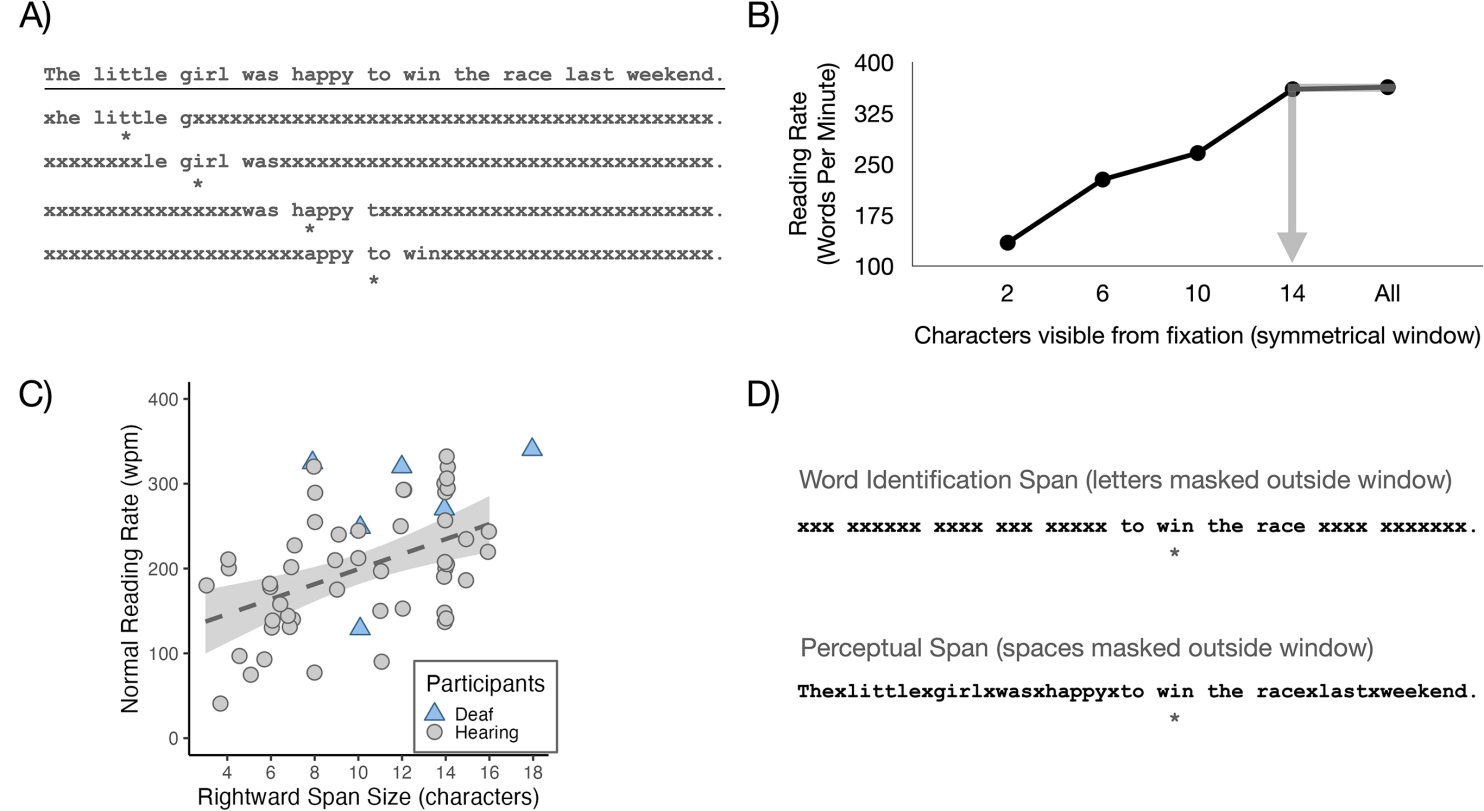
(**A**) Example of the moving window paradigm with the top line representing the full text and the lines beneath representing the text displayed during each successive fixation, represented by an asterisk (*). Note that only a single sentence on one line of text was presented during the experiment. (**B**) The size of the reading span as indicated by the window at which reading rate asymptotes (adapted from Rayner & Bertera, 1979). (**C**) Positive relationship between the rightward span size and reading rate based on data from 55 samples from the prior literature (values estimated when only figures were provided). (**D**) Examples of the manipulations used to separately assess the sizes of the perceptual span and word identification span.

The reading span is measured using the *moving window paradigm* (McConkie & Rayner, 1975), an eye tracking paradigm in which the text around the reader's fixation is revealed and is otherwise replaced with a mask (Figure 1A). The visible window of text moves instantaneously with the reader's fixation location, and the size is varied on different trials to measure reading rate (words per minute [wpm]) for different amounts of visible text. The window at which reading rate asymptotes (i.e., significantly increases from smaller windows but does not significantly increase with larger windows) is taken to indicate the size of the span (Figure 1B). In general, faster readers have larger spans than slower readers (Rayner et al., 2010; Sperlich, Meixner, & Laubrock, 2016). In fact, among 55 samples from the prior literature (including college students, children, older adults, second-language readers, and deaf signers), there is a significant positive relationship between span size and reading rate (*b* = 9.491, *SE* = 2.30, *t* = 4.12, *p* < 0.001) (Figure 1C).

To understand the mechanism by which larger spans support faster reading, it is critical to measure the perceptual span and the word identification span separately because they may contribute to reading rate in different ways (Morris, Rayner, & Pollatsek, 1990). A common interpretation of the role of the (rightward) reading span is to perceive the orthographic forms of upcoming words to initiate word identification earlier; we consider this to be the role of the word identification span, although this term is not always used (see Schotter et al., 2024). Alternatively, readers may use the span to perceive the spatial layout of the text; we consider this to be the role of the perceptual span, although this term is used more broadly in the literature to cover all types of reading spans (see Rayner, 2014). To assess the two spans independently, Schotter et al. (2024) used two variations of the moving window paradigm (Figure 1D). They assessed the perceptual span by masking only the spaces between the words outside of the visible window, and they assessed the word identification span by masking only the letters within the words outside of the visible window. This perceptual span manipulation disrupted the ability to perceive the spatial layout of the text but allowed for perception of linguistic information because the letters were visible outside of the window. The word identification span manipulation disrupted the ability to perceive linguistic information but allowed for perception of the spatial layout because the spaces between the words were still visible outside of the window. Supporting conclusions from past studies (Rayner, 1975; Rayner, 1998; Rayner, 2014; Rayner, Well, Pollatsek, & Bertera, 1982; Underwood & McConkie, 1985), Schotter et al. (2024) found that readers have a wider perceptual span (∼14 characters) than word identification span (∼7 characters), but they did not assess the relative contributions of the different spans to reading efficiency.

By comparing how strongly the two types of spans influence reading rate, we may shed light on longstanding debates over eye movement control in reading—for example, whether (implicit) decisions about whether and for how long to fixate a word are controlled by language processing or oculomotor constraints (see Reingold, Reichle, Glaholt, & Sheridan, 2012). A stronger influence of the word identification span would align more with *processing theories* of reading, which assume that fixation durations are determined by the ease of word identification (e.g., Just & Carpenter, 1980). In contrast, a stronger influence of the perceptual span would be more compatible with *oculomotor theories* of reading that assume that eye movements are programmed based on low-level features of text such as the locations of words and spaces (e.g., O'Regan, 1992).

If (parafoveal) language processing drives reading behavior, then a fast reading rate should be associated with high comprehension; therefore, it is important to take into account comprehension when investigating the impact of span size on reading rate. However, reading rate may have two possible relationships with reading comprehension, depending on how it is defined. For example, “(a) those individuals who tend to read the fastest also tend to comprehend the most, a positive between-individual relationship, and (b) when individuals increase their rate then the accuracy of comprehension decreases, a negative within-individual relationship” (Carver, 1990, p. 323). In other words, trait-level comprehension (e.g., score on a standardized test of reading comprehension ability) may be positively related with reading rate because better comprehenders can read faster, whereas state-level comprehension (e.g., accuracy in responding to comprehension questions within the experiment) may be negatively related with reading rate when an individual attempts to go faster than their most efficient rate.

There is no perfect measure of reading comprehension, and the various standardized tests of reading comprehension only moderately correlate with each other (Keenan & Meenan, 2014; Lee, Godwin, & Drieghe, 2024; Mézière, Yu, Reichle, Von Der Malsburg, & McArthur, 2023). In the current study, we used the Peabody Individual Achievement Test–Revised (PIAT-R) (Dunn & Markwardt, 1989) because, in contrast to tests that focus on whole passage reading and are more strongly related to listening comprehension, the PIAT-R assesses single sentence reading and is related to variability in word recognition skills during reading (Keenan, Betjemann, & Olson, 2008). Therefore, this measure is more likely to reflect aspects of reading comprehension that might relate to the distribution of visual attention reflected by the spans. In addition, the PIAT-R test is appropriate for use with deaf readers because it involves reading silently, rather than reading aloud, and it has been routinely used in studies comparing deaf and hearing readers (e.g., Bélanger et al., 2012; Bélanger et al., 2013; Belanger et al., 2018; Cooley et al., 2025a; Cooley et al., 2025b; Emmorey et al., 2025; Hirshorn, Dye, Hauser, Supalla, & Bavelier, 2022; McQuarrie & Abbott, 2013; Morere, 2012; Schotter et al., 2024; Sinclair et al., 2026; Stringer et al., 2024). Carver (1990) argued that using measures of reading comprehension ability that are timed “often reflect speed as much as accuracy” (Carver, 1990, p. 45), suggesting that finding a positive relationship between comprehension ability and reading rate requires measuring reading comprehension ability with an untimed assessment, separate from the text used to measure reading rate. However, most studies investigating this relationship have used comprehension measures of the text being read and show no relationship between reading rate and text comprehending accuracy (*r* = 0, *p* = 0.995) (Kuperman, Kyröläinen, Porretta, Brysbaert, Yang, 2021; see also Brysbaert, 2019a; Carver, 1990). It has been argued that this null relationship could be due to individual differences and the aggregation across many underlying relationships. For example, Brysbaert (2019a) noted that the relationship between reading rate and online text comprehension could be (1) negative, whereby some readers take more time to achieve a better understanding of the text; (2) curvilinear, whereby individual readers have an *optimal* rate, and comprehension declines both above and below this rate; or (3) positive, whereby readers who are better comprehenders need less time to understand the text and demonstrate both increased speed and increased comprehension (i.e., efficiency).

If deaf signers demonstrate enhanced reading efficiency, then they should show a positive relationship between reading comprehension ability and reading rate. Furthermore, if their wider spans are the source of their efficiency, then span size should also be positively related to both reading rate and comprehension. Schotter et al. (2024) found that deaf signers read more efficiently (i.e., faster with equivalent comprehension), but the two groups had equivalent sizes of the two spans. However, they did not investigate individual differences, so it is unclear whether deaf signers show a positive relationship between reading rate and comprehension ability and, if so, whether this is related to the sizes of either of the two spans.

Comparing the contribution of the two types of spans may help to understand the source of certain deaf signers’ reading efficiency: early deafness (Bavelier et al., 2001; Bosworth & Dobkins, 2002; Dye, Hauser, & Bavelier, 2009; Proksch & Bavelier, 2002) and/or sign language experience (Dye, Seymour, & Hauser, 2016; Stoll & Dye, 2019). For example, if deaf signers’ faster reading rates are more strongly related to their perceptual spans, it would suggest the source of their efficiency derives from early deafness, which is known to enhance visual attention to non-linguistic stimuli in the periphery (e.g., Bavelier et al., 2001; Proksch & Bavelier, 2002; Shiell, Champoux, & Zatorre, 2014). In contrast, if efficient deaf signers’ reading rates are more related to the word identification span, it would suggest that the source of their efficiency derives from sign language experience, which requires distributing visual attention to process *visual language* (Bosworth, Hwang, & Corina, 2022; Emmorey, Thompson, & Colvin, 2009; Lieberman, Hatrak, & Mayberry, 2014).

In the current study, we investigated the relationships between an individual's reading rate, the sizes of their two spans, and their reading comprehension ability (using an untimed standardized test), as well as how these relationships differed for deaf signers and hearing readers using a linear regression analysis. We expected that some findings would generalize to both groups—for example, that reading rate would be positively related to the size of the span (Rayner et al., 2010) (Figure 1c)—but a novel contribution of our study is that we compared the magnitude of this relationship between the two spans. A stronger relationship between reading rate and the word identification span would suggest that reading efficiency derives from parafoveal *linguistic* processing, a finding that would support processing theories of eye movement control in reading. Furthermore, if this relationship were stronger for deaf readers, it would support the idea that their efficiency derives from experience with extrafoveal linguistic processing (i.e., sign language) more so than being a consequence of deafness per se. Although past research has found no relationship between reading rate and text comprehension accuracy (Kuperman et al., 2021), it is unclear whether a null relationship would also be found for reading comprehension measured by an untimed standardized assessment. If, as Carver (1990) suggested, better comprehenders read faster, we should find a significant positive relationship between reading rate and (untimed) reading comprehension ability. Furthermore, deaf signers may be more likely to show such a positive relationship between reading comprehension skill and reading rate if their word identification span has a more functional role in their reading efficiency by virtue of their experience with parafoveal language processing.

## Methods

### Participants

Data were collected from 185 participants (65 deaf signers, 120 hearing non-signers) between the ages of 18 and 52, who had normal or corrected-to-normal vision, were proficient English readers, and had no history of speech/language or cognitive impairments. Deaf signers were prelingually and profoundly deaf (loss of ≥ 70 dB), used American Sign Language (ASL) as a primary means of communication, and were exposed to sign by age 7.^1^ Hearing non-signers were native English speakers with normal hearing.

### Materials, design, equipment, and procedure

Eye movements were tracked using an EyeLink 1000 Plus eye tracker (SR Research, Ottawa, ON, Canada) in a desktop setup (1000 Hz) with a chin and headrest to minimize head movements. Sentences consisted of 11 to 19 words (maximum of 86 characters) presented on a single line using the moving window paradigm (McConkie & Rayner, 1975) in two separate sets of blocks to test either the perceptual span or the word identification span. For the perceptual span, the window contained four characters to the left and six, 10, 14, 18, or 22 characters to the right; otherwise, the spaces were masked with x’s, but the letters remained intact. For the word identification span, the window contained four characters to the left and four, six, eight, or 10 to the right; otherwise, the letters were masked with x’s, but the spaces were preserved (see Figure 1D). There were also two blocks of normal sentences (i.e., without a moving window manipulation).

Participants consented to participate in the study in accordance with the Institutional Review Boards at four universities in the United States. Participants answered a questionnaire about demographic information and knowledge of languages other than English. Deaf participants also answered questions about their age at the time of first ASL exposure or usage, hearing level, and cochlear implant or hearing aid use. Communication was in ASL with the deaf participants and was in English with hearing participants. The eye tracker was calibrated using a three-point calibration procedure requiring each point to have an error less than 0.3 degrees of visual angle.

Trials were presented in blocks of increasing window size within each span manipulation, and the perceptual span blocks were always presented first. Each sentence was shown only once in the same window size for each participant, and the order of the sentences was randomized within a block. Twenty sentences were read in each block. On each trial, the participant looked at a fixation point in the center of the screen until the experimenter started the trial. A gaze box appeared on the left side of the screen, at the location of the start of the sentence. When the participant fixated the box, the sentence appeared in black Courier New 14-point font on a gray background. The participant read the sentence silently for comprehension and answered yes/no comprehension questions after 25% of trials to ensure that they were paying attention.

### Measures

*Reading rate* was measured in the normal text conditions. To assess the reliability of this measure, following Brysbaert (2019b), we used a linear mixed-effects model predicting reading rate in the full window condition by random intercepts for participants to estimate intraclass correlation coefficients (ICCs) using the icc() function in the R *performance* package (R Foundation for Statistical Computing, Vienna, Austria) (Lüdecke, Ben-Shachar, Patil, Waggoner & Makowski, 2021), which were 0.75 (range, 0.47–0.82) and 0.51 (range, 0.43–0.60) for the deaf signers and hearing non-signers, respectively.

*Span size* was estimated for each individual following an approach reported by Sperlich et al. (2016) and Meixner & Laubrock (2024) in which we fit a nonlinear mixed model (NLMM) and estimated the window size at which reading rate reached 95% of the asymptote (normal reading rate). The advantage of this approach is that it provides a point estimate, independent from the window sizes tested in the experiment, with precision to fractions of a character space. We used the nlsList(), SSasympOrig(), and nlme() functions in the R *nlme* package in R (version 3.1-164) (Pinheiro et al., 2026) to fit an asymptotic curve (i.e., the linear rate of change [lrc] toward an asymptote) to trial-level reading rate as a function of window size. We used the formula span = –log(1 – 0.95)/exp(lrc) to determine the point at which reading rate reached 95% of its asymptotic maximum.

*Reading comprehension ability* was measured using the 40 most difficult items from the PIAT-R (test–retest reliability *r* = 0.88) (Dunn & Markwardt, 1989). In this test, participants read a sentence and then, after it was removed, they selected one of four pictures that matched the meaning of the sentence. The items increased in difficulty, such that later sentences were longer and contained lower frequency words, more abstract concepts, and more complex syntax. Therefore, this test was a holistic assessment of sentence reading and comprehension ability. Because observing a positive relationship that reflects efficiency requires an untimed standardized assessment of comprehension, separate from the text used to measure reading rate (Carver, 1990), during administration of the test the participants’ eye movements were not monitored, nor was there mention of or emphasis on speed of completion. Items were scored until the participant made five errors within seven items, at which point the last incorrect item was counted as the ceiling item, and the number of correct answers prior to this were counted as the final score. We assessed the reliability of this measure in our samples by computing the ICC using the icc() function in the R *performance* package (Lüdecke, et al., 2021) based on a mixed model predicting item-level accuracy with item as a fixed effect and random intercepts and item slopes for participants. This ICC estimate, therefore, reflects the between-participant variance while accounting for increasing difficulty across items in the assessment. The resulting ICC estimates were 0.44 (range, 0.28–0.61) for the deaf group and 0.34 (range, 0.25–0.41) for the hearing group.

#### Background measures

We also measured spelling ability (spelling recognition; test–retest reliability *r* = 0.93) (Andrews & Hersch, 2010), non-verbal intelligence (Kaufman Brief Intelligence Test, Second Edition [KBIT-2]; test–retest reliability *r* = 0.85) (Kaufman & Kaufman, 2004), sign comprehension ability (American Sign Language Comprehension Test [ASL-CT]; internal reliability α = 0.83) (Hauser et al., 2016), age, and years in college or higher education.

#### Data exclusions and statistical power

Participants were excluded if they had a PIAT score below the starting point (one deaf participant), experienced motion sickness during the experiment (three hearing participants), had fewer than 10 usable trials in any condition in the experiment (four deaf and three hearing participants), or if the NLMM model could not produce a span estimate (10 deaf and five hearing participants).^2^ These exclusions led to a final dataset of 50 deaf signers and 109 hearing non-signers. The mean age of ASL acquisition for the included deaf signers was 1.3 years (*SD* = 1.79).

Our sample sizes are similar to those of past studies that have detected significant differences in span size based on reading skill with 44 participants (Sperlich et al., 2016; Veldre & Andrews, 2014) or 70 participants (Choi, Lowder, Ferreira, & Henderson, 2015). In addition, we conducted a sensitivity analysis using G*Power to compute the effect size we would be able to detect in a linear multiple regression with α = 0.05, power = 0.8, and six predictors for our smaller sample (i.e., *n_deaf_* = 50). This analysis revealed that our sample size should be sufficient to detect an effect size (*f*^2^) as small as 0.314.

## Results

The data and code used for the analyses are available on the Open Science Framework (OSF) at https://osf.io/r632v/?view_only=f77c93df3da3406c9c44e79b6ac79083.

### Group comparisons

The deaf signers read significantly faster than the hearing non-signers (*p* < 0.005), but the groups did not differ in reading comprehension ability, the sizes of the spans, spelling ability, or nonverbal intelligence (all *p* > 0.07) (Table 1). The hearing non-signers were significantly younger and had fewer years of education due to a larger proportion of current college students in the sample. The hearing non-signers had slightly higher accuracy on the comprehension questions, but both groups performed above 90% correct, well above “the traditional 75% criterion used to define what it means to comprehend accurately” (Carver, 1990, p. 27). Only the deaf signers were tested on sign language comprehension ability, and they scored similarly to the normative samples of early deaf signers reported in Hauser et al. (2016) and Sehyr and Emmorey (2022).

**Table 1.** Descriptive statistics for the groups.

	Deaf signers (*n* = 50)			Hearing non-signers (*n* = 109)			
Variable	*M*	*SD*	Range	*M*	*SD*	Range	*p* (*t*-test)
Normal reading rate	325	134.0	169–745	261	64.7	144–491	**<0.005**
Reading comprehension ability (PIAT-R)	84.5	10.3	63–99	86.2	9.2	61–97	0.32
Comprehension accuracy (% correct)	90.9	6.8	72.7–100	94.5	4.7	720.7–100	**<0.005**
Word identification span	9.2	3.2	5.7–21.8	9.0	2.4	50.2–150.8	0.63
Perceptual span	11.0	3.9	4.9–29.2	10.3	2.7	60.0–190.7	0.25
Spelling ability (recognition)	73.3	9.2	47–87	73.8	7.1	54–86	0.78
Nonverbal intelligence (KBIT)	38.5	5.2	23–46	38.3	3.9	24–46	0.79
Age	32.8	8.8	18–52	26.6	9.3	18–51	**<0.001**
Years in college	5.4	3.5	0–15	3.6	2.7	0–13	**<0.005**
Sign language comprehension (ASL-CT)	25.5	2.6	16–28	—	—	—	—

*Notes:* The *p* values come from Welch's independent samples *t*-tests. Possible ranges for the test scores were 60 to 100 (PIAT-R), 0 to 87 (spelling), 15 to 46 (KBIT), and 0 to 30 (ASL-CT). Boldface indicates statistical significance.

### Simple correlations between variables

The simple correlations between the variables for deaf signers and hearing non-signers can be seen in Tables 2 and 3, respectively. Although reading rate was not correlated with comprehension accuracy for either group (both *p* > 0.05), it was positively correlated with reading comprehension ability for deaf signers (*r* = 0.40, *p* < 0.01) but not hearing non-signers (*r* = –0.06, *p* > 0.05) (see Table 4 for a statistical analysis testing the interaction with group). The finding that deaf, but not hearing, readers who are better comprehenders read faster suggests that reading rate is an indicator of reading efficiency only for deaf signers. The perceptual span was only weakly correlated with the word identification span (*r* = 0.19, *p* > 0.05 and *r* = 0.21, *p* < 0.05 for deaf signers and hearing non-signers, respectively^3^), further supporting the distinction between the two types of spans.

**Table 2.** Correlations of variables for deaf signers.

Variable	1	2	3	4	5	6	7	8	9
1. Normal reading rate	—	—	—	—	—	—	—	—	—
2. Reading comprehension ability	0.40**	—	—	—	—	—	—	—	—
3. Comprehension accuracy	−0.01	0.42**	—	—	—	—	—	—	—
4. Word identification span	0.65***	0.33*	0.10	—	—	—	—	—	—
5. Perceptual span	0.20	−0.08	0.03	0.19	—	—	—	—	—
6. Spelling ability	0.46***	0.62***	0.48***	0.40**	−0.04	—	—	—	—
7. Nonverbal intelligence	0.19	0.49***	0.48***	0.33*	0.16	0.51***	—	—	—
8. Age	−0.37**	0.05	0.09	−0.31*	−0.36**	−0.02	−0.10	—	—
9. Years in college	0.21	0.25	0.17	0.09	−0.08	0.31*	0.16	0.35*	—
10. ASL comprehension	−0.14	0.14	0.37**	−0.12	0.09	0.10	0.37*	0.00	−0.14

*Notes:* *p < 0.05, **p < 0.01, ***p < 0.001.

**Table 3.** Correlations of variables for hearing non-signers.

Variable	1	2	3	4	5	6	7	8
1. Normal reading rate	—	—	—	—	—	—	—	—
2. Reading comprehension ability	−0.06	—	—	—	—	—	—	—
3. Comprehension accuracy	0.01	0.37***	—	—	—	—	—	—
4. Word identification span	0.45***	0.03	0.12	—	—	—	—	—
5. Perceptual span	0.34**	−0.03	−0.10	0.21*	—	—	—	—
6. Spelling ability	0.14	0.23*	0.40***	0.07	−0.06	—	—	—
7. Nonverbal intelligence	0.09	0.42***	0.31***	−0.08	−0.10	0.22*	—	—
8. Age	−0.08	0.23*	0.19*	0.08	0.06	0.23*	0.14	—
9. Years in college	0.09	0.07	0.20*	0.05	0.08	0.23*	0.11	0.64***

*Notes:* *p < 0.05, **p < 0.01, ***p < 0.001.

**Table 4.** Results of linear regressions predicting reading rate as a function of group, reading comprehension ability, and span sizes, including covariates for accuracy in the experiment, age, and years in college.

	Full model				Deaf signers				Hearing non-signers			
Predictor	Est.	*SE*	*t*	*p*	Est.	*SE*	*t*	*p*	Est.	*SE*	*t*	*p*
Intercept	293.04	6.08	48.21	**<0.001**	325.33	13.04	24.96	**<0.001**	261.49	5.30	49.36	**<0.001**
Group	−56.35	13.45	−4.19	**<** **0.001**	—	—	—	—	—	—	—	—
Reading comprehension ability	17.46	6.54	2.67	**<** **0.01**	39.46	15.75	2.51	**<** **0.05**	−1.08	5.91	−0.18	0.86
Word identification span size	16.02	2.19	7.30	**<** **0.001**	18.95	4.67	4.06	**<** **0.001**	10.99	2.31	4.77	**<** **0.001**
Perceptual span size	4.26	1.83	2.32	**<** **0.05**	2.06	3.70	0.56	0.58	6.09	2.07	2.94	**<** **0.01**
Accuracy in the experiment	−11.35	6.38	−1.78	0.08	−25.97	14.66	−1.77	0.08	−0.47	5.94	−0.08	0.94
Age	−2.66	0.76	−3.52	**<** **0.005**	−4.24	1.82	−2.33	**<** **0.05**	−1.83	0.77	−2.37	**<** **0.05**
Years in college	7.42	2.24	3.31	**<** **0.005**	8.93	4.21	2.12	**<** **0.05**	5.15	2.58	2.00	**<** **0.05**
Group × reading comprehension	−27.48	12.17	−2.26	**<** **0.05**	—	—	—	—	—	—	—	—
Group × word identification span	−8.86	4.44	−2.00	**<** **0.05**	—	—	—	—	—	—	—	—
Group × perceptual span	2.89	3.71	0.78	0.44	—	—	—	—	—	—	—	—
Observations	159				50				109			
*R* ^2^/*R*^2^ adjusted	0.523/0.490				0.583/0.525				0.310/0.270			

Significant effects (at the *p* < 0.05 level or lower) are indicated in bold.

### Predicting reading rate by the different reading spans and comprehension ability

We used a linear regression to predict reading rate by group, reading comprehension ability, the sizes of the two spans, and the interactions between group and the other variables, with covariates for age, years of education, and accuracy on the in-experiment comprehension questions. All variables were centered: Group was entered with a sum-to-zero contrast, and reading comprehension ability and in-experiment accuracy were *z*-scored (i.e., scaled to standard deviation from the mean), but the other variables were not scaled so the effect the span estimates represents the effect of increasing the span by one character and the effects of age and years in college represent the effect of increasing by 1 year. The variance inflation factors (VIFs) of the predictors in this model were all low (<1.76), suggesting that multicollinearity was not a problem. We also performed separate analyses to determine the magnitude of these effects for each group.

In the full model, there were significant effects of group, both span sizes, and reading comprehension ability, as well as age and years in college. Furthermore, there were significant interactions between group and both reading comprehension ability and the size of the word identification span: larger effects for the deaf signers than hearing non-signers.

**Figure 2.**
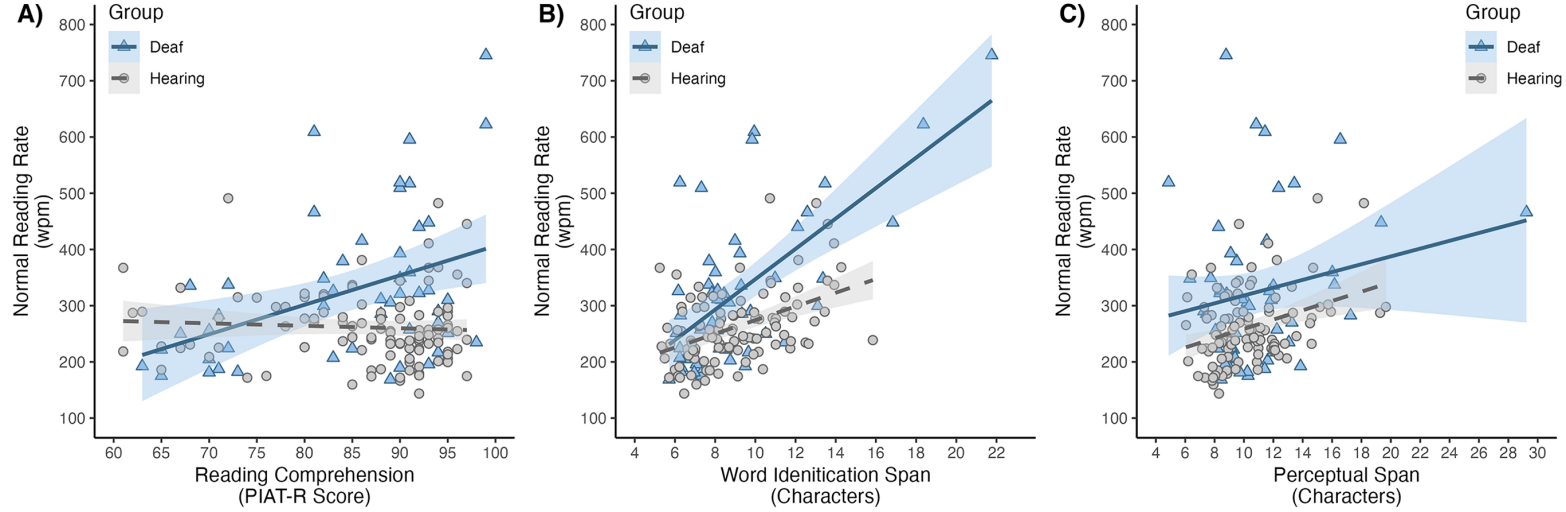
(**A–C**) Scatterplots of relationships between reading rate and reading comprehension ability (**A**), the word identification span (**B**), and the perceptual span (**C**) for deaf signers (blue triangles) and hearing non-signers (gray circles). Note that the shaded areas represent 95% confidence intervals around the linear best fit.

In the separate analyses, reading comprehension ability was a significant predictor of reading rate for deaf signers but not hearing non-signers (Figure 2A), and the interaction between reading comprehension ability and group was significant in the full model (Table 4). The word identification span size was a significant predictor of reading rate for both groups, but the effect size was larger for deaf signers (Figure 2B), and the interaction between the word identification span and group was significant in the full model (Table 4). The effect size for the perceptual span size was small for both groups, and was only statistically significant for hearing non-signers, but not for deaf signers (Figure 2C), although the interaction between the perceptual span and group was not significant in the full model (Table 4). With respect to the covariates for both groups, there was no effect of accuracy in the experiment, age was a significant negative predictor (older people read slower), and education was a significant positive predictor (people with more years of education read faster).

In response to a reviewer, we ran an analysis to test whether the effects reported above hold after excluding three deaf participants with word identification spans higher than the range for the hearing participants (i.e., “outliers” with word identification spans of 16 characters or larger). This analysis revealed the exact same patterns of significance as the analyses reported in Table 4, except that the interaction between group and the word identification span was no longer significant (see Supplementary Appendix). However, this result should be expected because the main finding is that readers with larger word identification spans read faster and this relationship is stronger for deaf signers. Therefore, this effect would be reduced by excluding the deaf participants with the largest spans (and also excluding observations also reduces statistical power).

## Discussion

Our investigation of individual differences in reading efficiency revealed several generalizable conclusions that apply to both hearing and deaf readers, but these relationships differed in magnitude between deaf signers and hearing non-signers. Specifically, for all readers, the word identification span—more than the perceptual span—had a positive influence on reading rate. However, the effect of the word identification span on reading rate was significantly stronger for deaf signers, who read faster than hearing readers with equivalent offline comprehension abilities. These findings suggest that overall, but especially for deaf readers, parafoveal perception of linguistic information is more critical to reading efficiency than perception of the spatial layout of the text. Such a finding aligns with processing theories of reading (Just & Carpenter, 1980; Reingold et al., 2012) to suggest that ongoing language processing is the primary determiner of eye movement control and the speed with which a reader can progress through the text. The perceptual span was weakly related to reading rate, and this relationship was only significant for hearing non-signers, suggesting that the latter group relies more on the visual properties of text to modulate their reading speed. We take this to suggest that individual differences in reading rates are more associated with successful linguistic processing and comprehension skills for deaf readers compared with hearing readers. Offline reading comprehension ability (but not online comprehension accuracy in the experiment) was a significant predictor of reading rate for deaf signers, but there was no relationship between either comprehension measure and reading rate for hearing readers. These findings support the idea that deaf signers’ efficiency derives from an ability to identify words outside of their fixation location, and to use this mechanism to support both reading rate and comprehension. In contrast, hearing readers use both the word identification span (to a lesser degree) and the perceptual span (to a greater degree) to support faster reading rates, but their reading speed is not associated with greater online or offline comprehension skill.

The significant relationship between the size of the perceptual span and reading rate for hearing readers (a relationship that was weaker and non-significant for deaf signers) indicates they attend more to visuospatial aspects of parafoveal text. This pattern suggests that their reading process draws more on the mechanisms proposed by oculomotor models of eye movement control in reading (e.g., O'Regan, 1992). Furthermore, the null relationship between reading comprehension ability and either of the reading spans suggests that hearing people may rely less on parafoveal language processing for comprehension. Rather, hearing people may depend on parafoveal information obtained within their spans primarily to maintain a fast reading rate and not to comprehend. This idea is consistent with the null relationships between both online and offline measures of comprehension and reading rate for hearing readers (see also Brysbaert, 2019a; Kuperman et al., 2021). One possibility is that hearing peoples’ comprehension may be more associated with foveal processing efficacy. We did not assess foveal processing in this study, but several studies using moving mask manipulations to prohibit foveal processing show that doing so not only slows reading but also interferes with readers’ ability to accurately report the words in the text (Rayner & Bertera, 1979) or to answer comprehension questions about it (Rayner, Yang, Schuett, & Slattery, 2014). Alternatively, it has long been theorized that successful reading requires two skills (e.g., the simple view of reading) (Hoover & Gough, 1990): word decoding and language comprehension (typically defined as listening comprehension). The size of the word identification may be more related to the word decoding mechanism and therefore the initial speed with which a person can read, whereas comprehension of the text may be more related to processes that (hearing) people engage to make sense of the speech-based representations after they have decoded the text.

Both groups showed a significant positive relationship between the size of the word identification span and reading rate, suggesting that the ability to initiate word recognition in the parafovea allows people to read faster. It is therefore notable that we did not observe any hearing participants with a word identification span of 16 characters or larger, nor who read at a rate of 492 wpm or faster, especially considering that we deliberately tested a large sample of hearing participants, spanning a large age and education range. These ranges, and the group means, align well with the published literature on these variables, suggesting that it is not that our group of hearing participants represent a restricted (i.e., low) range within the normal population, but rather that there are indeed some deaf signers who are particularly efficient readers (because of their wide word identification spans). Furthermore, only the deaf signers showed a significant positive relationship between the size of the word identification span and comprehension ability, and between reading rate and reading comprehension ability (see also Bélanger et al., 2012; Bélanger et al., 2018; Emmorey et al., 2025; Stringer et al., 2024). These relationships support the idea that deaf signers who are better comprehenders of English text utilize their word identification spans to a greater degree. Furthermore, the fact that the size of the word identification span was related to both reading rate and comprehension ability suggests that the source of the deaf signers’ efficiency is their ability to extract linguistic information far from fixation in a way that both supports speed and comprehension of the text. This may explain why deaf signers’ high reading rate has been specifically linked to a higher rate of skipping words (Bélanger et al., 2013; Cooley et al., 2025a; Cooley et al., 2025b; Emmorey et al., 2025; Schotter et al., 2024; Sinclair et al., 2026; Traxler et al., 2021) and a lower rate of regressing back to reread them (Bélanger et al., 2013; Bélanger et al., 2018; Cooley et al., 2025b; Emmorey et al., 2025; Schotter et al., 2024). If deaf signers can recognize words more effectively from parafoveal vision (i.e., within the word identification span) then they may not need to directly fixate them to understand them in the way that hearing readers do.

The fact that deaf signers’ word identification span showed a stronger relationship than the perceptual span to both reading rate and comprehension ability suggests that their reading efficiency may arise from their sign language experience, rather than deafness per se. Support for this idea comes from the recent finding that hearing native signers also exhibit a larger (leftward) word identification span compared with hearing non-signers (Emmorey et al., 2025). Sign language may be responsible for this relationship because, like text, it allows for the simultaneous perception of linguistic elements across the visual field. For example, during sign conversation, foveal vision is used to perceive grammatically informative facial expressions while parafoveal vision is used to perceive manual signs (Bosworth et al., 2022; Emmorey et al., 2009). Additionally, when learning sign language, children constantly shift their fixation location between the sign and the referent (Lieberman et al., 2014), whereas shifting visual attention is unnecessary when mapping a spoken label to a visual referent. Thus, multiple visual–linguistic elements are simultaneously accessible during reading in a way that is more analogous to sign comprehension than it is to listening to speech. Furthermore, deaf people who do not use sign language (i.e., “oral” deaf) or who learned it later in life are not more efficient readers (Coulter & Goodluck, 2015; Gómez-Merino, Fajardo, Ferrer, & Arfé, 2020; Gómez-Merino, Fajardo, Ferrer, & Joseph, 2022; Tomasuolo, Roccaforte, & Di Fabio, 2019; Traxler, Corina, Morford, Hafer, & Hoversten, 2014), and they engage in parafoveal processing of speech-based representations of print similarly to hearing readers (Blythe, Dickins, Kennedy, & Liversedge, 2018). These findings suggest that they rely on the same reading processes but are less effective at doing so, perhaps because they do not benefit from the visual language processing advantages afforded by sign language and/or because they are more likely to experience language deprivation early in life, at a time that is critical for cognitive and language development (Hall, Hall, & Caselli, 2019; Humphries et al., 2012; Humphries, Mathur, Napoli, Padden, & Rathmann, 2022).

In summary, readers’ word identification span (and, to a lesser degree, their perceptual span) is related to their reading rate, but only for deaf signers was reading comprehension ability related to the word identification span or reading rate. These findings suggest that hearing readers rely on mechanisms proposed in both processing and oculomotor theories of reading to support reading speed and different mechanisms to understand the text. In contrast, deaf early signers’ reading efficiency is due to an ability to extract specifically linguistic information from the parafovea, aligning more with mechanisms proposed in processing theories of reading. Together, these data suggest a critical role for visual language processing in reading efficiency, highlighting the importance of the vision–language interface in performing complex everyday tasks such as understanding a text.

## Supplementary Material

Supplement 1

## Footnotes

1 Of the deaf participants included in the analyses, most learned sign language at birth (*n* = 29) or by age 4 (*n* = 18), and very few later than that (*n* = 3).

2 Spans could not be estimated when readers had essentially flat reading rates across window size conditions.

3 The correlation for the combined sample was *r* = 0.20 and *p* < 0.01.
