# Supplementary material for: Identifying the visual and linguistic sources of reading efficiency: A comparison of individual differences among deaf and hearing readers
Source: J Vis. 2026 Jun 1;26(6):2. doi: 10.1167/jov.26.6.2 (PMC13235764; doi:10.1167/jov.26.6.2)
Supplement: Supplement 1 [file jovi-26-6-2_s001.docx]

**Appendix**

*Table A1.* Results of linear regressions predicting reading rate as a function of group, reading comprehension ability, and span sizes, including covariates for accuracy in the experiment, age, and years in college after excluding three deaf participants with word identification spans of 16 characters or larger.

|  | **Subset Model** | | | | **Deaf Signers  excluding WIS > 16** | | | | **Hearing Non-signers** | | | |
| --- | --- | --- | --- | --- | --- | --- | --- | --- | --- | --- | --- | --- |
| *Predictors* | *Est.* | *SE* | *t* | *p* | *Est.* | *SE* | *t* | *p* | *Est.* | *SE* | *t* | *p* |
| (Intercept) | 287.63 | 6.19 | 46.49 | **<0.001** | 307.46 | 13.38 | 22.99 | **<0.001** | 261.49 | 5.30 | 49.36 | **<0.001** |
| Group: Deaf vs. Hearing | -51.61 | 13.56 | -3.81 | **<0.001** |  |  |  |  |  |  |  |  |
| Reading Comprehension Ability (PIAT) | 17.79 | 6.48 | 2.75 | **0.007** | 40.00 | 15.46 | 2.59 | **0.013** | -1.08 | 5.91 | -0.18 | 0.855 |
| Word Identification Span (WIS) | 14.32 | 2.92 | 4.90 | **<0.001** | 16.03 | 7.05 | 2.27 | **0.028** | 10.99 | 2.31 | 4.77 | **<0.001** |
| Perceptual Span (PS) | 5.40 | 1.87 | 2.89 | **0.004** | 4.52 | 3.91 | 1.16 | 0.255 | 6.09 | 2.07 | 2.94 | **0.004** |
| Accuracy in the experiment | -12.20 | 6.40 | -1.91 | 0.059 | -29.00 | 15.18 | -1.91 | 0.063 | -0.47 | 5.94 | -0.08 | 0.937 |
| Age | -2.60 | 0.75 | -3.46 | **0.001** | -3.99 | 1.82 | -2.20 | **0.034** | -1.83 | 0.77 | -2.37 | **0.019** |
| Years in college | 7.39 | 2.24 | 3.31 | **0.001** | 8.96 | 4.26 | 2.10 | **0.042** | 5.15 | 2.58 | 2.00 | **0.049** |
| Group x PIAT | -27.99 | 12.01 | -2.33 | **0.021** |  |  |  |  |  |  |  |  |
| Group x WIS | -5.42 | 5.86 | -0.92 | **0.357** |  |  |  |  |  |  |  |  |
| Group x PS | 0.52 | 3.79 | 0.14 | 0.890 |  |  |  |  |  |  |  |  |
| Observations | 156 | | | | 47 | | | | 109 | | | |
| R^2^ / R^2^ adjusted | 0.391 / 0.349 | | | | 0.422 / 0.336 | | | | 0.310 / 0.270 | | | |

*Note*: The data from the hearing non-signers is the same as reported in the main text and is reproduced here for ease of comparison.
